# Dose-Dependent Responses of I3C and DIM on T-Cell Activation in the Human T Lymphocyte Jurkat Cell Line

**DOI:** 10.3390/ijms18071409

**Published:** 2017-07-01

**Authors:** Man Liu, Rumana Yasmeen, Naomi K. Fukagawa, Liangli Yu, Young S. Kim, Thomas T. Y. Wang

**Affiliations:** 1 Department of Biomedicine and Food Science, College of Life Science, Jiangsu Normal University, Xuzhou 221116, Jiangsu Province, China; liuman861214@163.com; 2 Diet, Genomics and Immunology Lab, Beltsville Human Nutrition Research Center, Agricultural Research Service (ARS), United States Department of Agriculture (USDA), Beltsville, MD 20705, USA; Rumana.Yasmeen@ars.usda.gov (R.Y.); Naomi.Fukagawa@ars.usda.gov (N.K.F.); 3 Department of Nutrition and Food Science, University of Maryland, College Park, , MD 20742, USA; Lyu5@umd.edu; 4 Nutritional Sciences Research Group, Division of Cancer Prevention, National Cancer Institute, NIH, Bethesda, MD 20892, USA; kimyoung@mail.nih.gov

**Keywords:** I3C, DIM, human T-lymphocytes, pro-inflammatory cytokines, NF-κB, NFAT1

## Abstract

Indole-3-carbinol (I3C) and its dimer diindolylmethane (DIM) are bioactive metabolites of a glucosinolate, glucobrassicin, found in cruciferous vegetables. Both I3C and DIM have been reported to possess pro-apoptotic, anti-proliferative and anti-carcinogenic properties via modulation of immune pathways. However, results from these studies remain inconclusive since they lack thorough evaluation of these bioactives’ physiological versus pharmacological effects. In the present study, we investigated I3C and DIM’s dose-dependent effects on cytokines production in human T lymphocytes Jurkat cell line (Clone E6-1). The results showed that I3C and DIM pretreatment, at higher concentrations of 50 and 10 μM, respectively, significantly increased PMA/ionomycin-induced interleukin-2 (IL-2), interleukin-8 (IL-8) and tumor necrosis factor-α (TNF-α) production, measured by real time polymerase chain reaction (RT-PCR) and enzyme linked immunosorbent assay (ELISA). As a plausible mechanism underlying such pronounced cytokine release, we found robust increase in downstream nuclear factor κB (NF-κB) and nuclear factor of activated T-cells 1 (NFAT1) signaling with I3C pretreatment, whereas DIM pretreatment only significantly induced NF-κB activation, but not NFAT1. We hypothesize that I3C/DIM pretreatment primes the T cells to become hyperresponsive upon PMA/ionomycin stimulation which in turn differentially induces two major downstream Ca^2+^-dependent inflammatory pathways, NF-κB and NFAT1. Our data show novel insights into the mechanisms underlying induction of pro-inflammatory cytokine release by pharmacological concentrations of I3C and DIM, an effect negligible under physiological conditions.

## 1. Introduction

T lymphocytes serve as a critical component of cell-mediated immunity, involved in the modulation of other immune cell function and destruction of cells infected with pathogens [1,2]. When activated, T-cells also produce a broad range of cytokines and chemokines that are important in immune cell recruitment and in regulation of inflammatory responses. Elevated levels of these inflammatory cytokines are associated in the pathophysiology of many chronic inflammatory diseases, including cancer [3]. Therefore, thorough understanding of the T-cell signaling can potentially identify new targets for therapeutic intervention in order to prevent or protect against aberrant immune responses.

T-cell receptor activation (TCR) causes a rapid increase in the intracellular Ca^2+^ level, which in turn regulates important cellular processes through the activation of several signaling molecules [4,5]. However, calcium alone can also trigger T-cell signaling and modulate cytokine production, bypassing TCR activation [6,7]. Mobilization of intracellular calcium can also activate nuclear factor κB (NF-κB), and nuclear factor of activated T cell (NFAT), the key transcription factors involved in inflammation. Moreover, both NF-κB, and NFAT are implicated in the activation of the immune and inflammatory responses in T lymphocytes and regulation of cytokine secretion, such as interleukin-2 (IL-2), IL-8, tumor necrosis factor-α (TNF-α) [8,9,10,11].

The NF-κB transcription factor is known to promote immunity through modulating induction of expression of genes and cytokines. It consists of two subunits, p50 and p65, which, in the resting state, are held in the cytoplasm by the inhibitory κB (IκB) proteins [12]. Phosphorylation of IκB proteins leads to its degradation allowing the NF-κB complex to translocate into the nucleus. Ser536-phosphorylated p65 is found predominantly in the cytosol and is a downstream event following IκB degradation [13]. In contrast, dephosphorylation of NFAT leads to its activation and translocation into the nucleus, where it mediates the regulation of various cytokines, including IL-2, IL-4, IL-8, TNF-α, and IL-3/granulocyte-macrophage colony-stimulating factor [12]. One of the five members of the NFAT family, NFAT1, is constitutively expressed in T-lymphocytes and has been reported to be associated with several immune diseases [14,15]. Therefore, modulation of NF-κB, and NFAT1 could serve as important therapeutic targets to counteract the inflammation pathways associated with chronic diseases.

In recent years, there has been a substantial public health interest in the use of natural products as an adjunct therapy to modulate immune responses and attenuate inflammatory processes. Cruciferous vegetables (i.e., broccoli, cabbage, brussels sprouts, cauliflower, etc.) are rich in sulfur-containing compounds, known as glucosinolates. Enzymatic breakdown of an indole glucosinolate, known as glucobrassicin, generates indole-3-carbinol (I3C). In the acidic environment of the stomach, I3C forms complex mixture of bioactive compounds, such as the dimer 3, 3’-diindolylmethane (DIM) [16]. I3C and DIM have gained substantial scientific attention due to their pro-apoptotic, anti-proliferative, and anti-angiogenic activities [17,18,19,20,21]. However, in the majority of existing studies concerning anti-inflammatory properties of I3C and DIM, high doses are often used, resulting in production of pharmacological rather than physiological effects [22,23]. Chemopreventive properties attributed to these phytochemicals seem to stem from induction of apoptosis and cell cycle arrest in a variety of cancer cells at supraphysiological doses [24]. Bioavailability studies in rodents have reported maximum plasma and tissue concentrations of 28 and 170 µmol/L (15 min) for I3C and 4 and 16 µmol/L (2 h) for DIM, respectively [25,26]. In humans, although information on bioavailability and tissue distribution of I3C and DIM is limited [27], two studies detected DIM in plasma at 2.5 µmol/L that peaked at 2 h following administration of I3C [28,29]. At present, I3C and DIM supplements are widely touted as an adjunct for prevention of various types of cancer, backed by many reported health effects that may be irrelevant to what can be physiologically achievable in vivo. Without conclusive evidence, such health claim could inflict greater harm to those at risk, prompting us to compare physiological versus pharmacological effects of I3C and DIM, their role in T-cell mediated immunity and possible mechanisms at play. In the present study, we investigated the dose-dependent effects of I3C and DIM pretreatment on the regulation of inflammatory responses upon activation of TCR signaling. Using Jurkat T-cells exposed to phorbol-12-myristate-13-acetate (PMA)/ionomycin (calcium ionophore) cocktail, we showed that I3C and DIM pretreatment, at high concentrations, can induce Ca^2+^-dependent downstream inflammatory pathways: I3C activates NF-κB and NFAT1 signaling, while DIM, unlike I3C, acting through NF-κB, led to elevated secretion of pro-inflammatory cytokines. Our results suggest that pharmacological doses of I3C and DIM primed the T-cells to become hyperresponsive to PMA/Ionomycin, which in turn augmented T-cell signaling and a robust induction of inflammatory mediators. These results provide critical information that raises the need for caution regarding the use of pharmacological concentrations of I3C and DIM to prevent inflammatory diseases.

## 2. Results

### 2.1. Effects of Indole-3-Carbinol (I3C)/Dimer Diindolylmethane (DIM) Concentrations on Cell Viability

**Figure 1:**
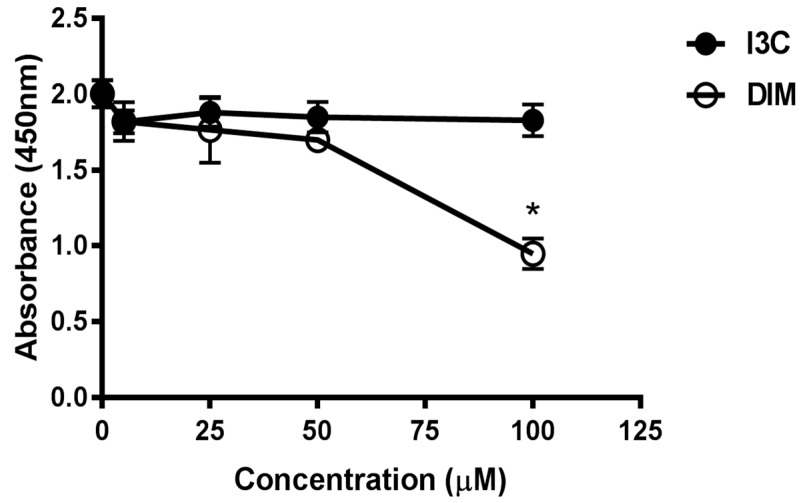
Assessment of cell viability following treatment with different doses of Indole-3-Carbinol (I3C) and Dimer Diindolylmethane (DIM) in Jurkat cells. Jurkat cells were plated at a density of 4 × 10^−5^ cells/mL in a 96 well plate overnight. Cells were treated with varying doses of I3C or DIM for 48 h and cell viability was assessed using CCK8 kit following manufacturer’s instructions. Results expressed as mean ± SD (*n* = 3) from three independent experiments. * indicates significantly different compared to control (0.1% dimethyl sulfoxide (DMSO) at *p* < 0.05 (One-Way Anova).

To evaluate the cytotoxic potential of various doses of I3C and DIM, we treated Jurkat cells with 5, 25, 50 and 100 µM of I3C or DIM for 48 h. Cell viability assays were then performed for CCK8. We observed minimal inhibition in proliferation with all doses of I3C and DIM, except 100 µM of DIM (Figure 1). These data helped us choose concentrations that do not affect cell viability for our subsequent experiments.

### 2.2. I3C and DIM Increase Interleukin-2 (IL-2), Interleukin-8 (IL-8) and Tumor Necrosis Factor-α (TNF-α) mRNA Levels in Activated T Cells

**Figure 2:**
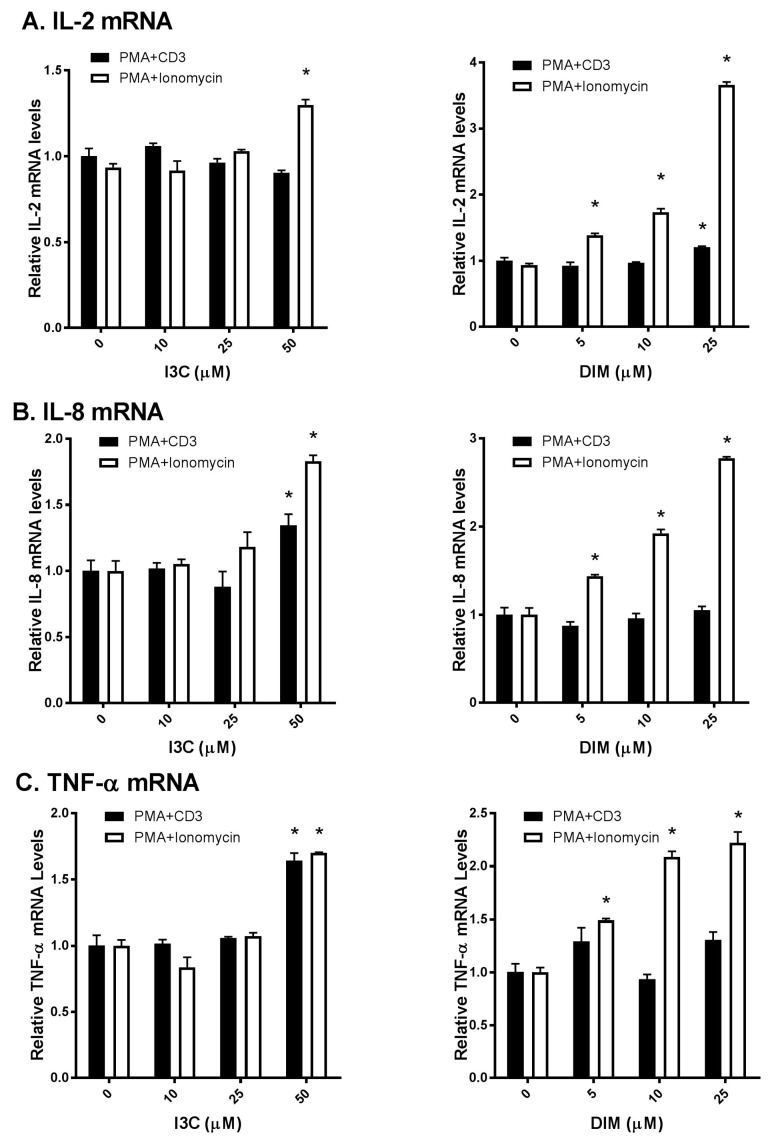
Effects of I3C, DIM on Interleukin-2 (IL-2), Interleukin-8 (IL-8) and Tumor Necrosis Factor-α (TNF-α) mRNA levels in Jurkat cells. Jurkat cells were treated with 10, 25 or 50 μM of I3C or 5, 10 or 25 μM of DIM for 48 h and then stimulated with phorbol-12-myristate-13-acetate (PMA) + anti-cluster of differentiation 3 (CD3) antibody or PMA + ionomycin for 6 h. Genes expression determinations of: (**A**) IL-2; (**B**) IL-8; and (**C**) TNF-α were analyzed using real time polymerase chain reaction (RT-PCR). Results expressed as mean ± SD (*n* = 3) from three independent experiments. * indicates significantly different from control at *p* < 0.05 (Two-Way Anova).

As T lymphocytes regulate a broad range of cytokines that in turn regulate active immune responses [1], we were interested to examine I3C and DIM’s effects on T-cells. We used commonly known stimulators of TCR signaling (PMA/anti-CD3 and PMA/ionomycin) to activate T-cells and compared I3C and DIM’s effects on inflammatory responses. Interestingly, we observed differential responses of I3C and DIM to these two combinations of T-cell activation. I3C, only at 50 µM, could modestly increase IL-2 mRNA expression upon PMA/ionomycin stimulation (Figure 2A); however, IL-2 mRNA expression, upon PMA/anti-CD3 induction, remained comparable to control (vehicle-treated).

In contrast, DIM exhibited a dose-dependent increase in IL-2 mRNA expression, only upon PMA/ionomycin stimulation. Similarly, I3C and DIM markedly increased PMA/ionomycin-mediated IL-8 and TNF-α expression, while minimal changes in IL-8 and TNF-α were observed upon PMA/anti-CD3 stimulation (Figure 2B,C). Of note, I3C, only at 50 µM, showed maximal induction of these pro-inflammatory cytokines, whereas DIM showed a dose-dependent effect.

### 2.3. I3C and DIM Increase IL-2, IL-8 and TNF-α Protein Levels in Activated T cells

**Figure 3:**
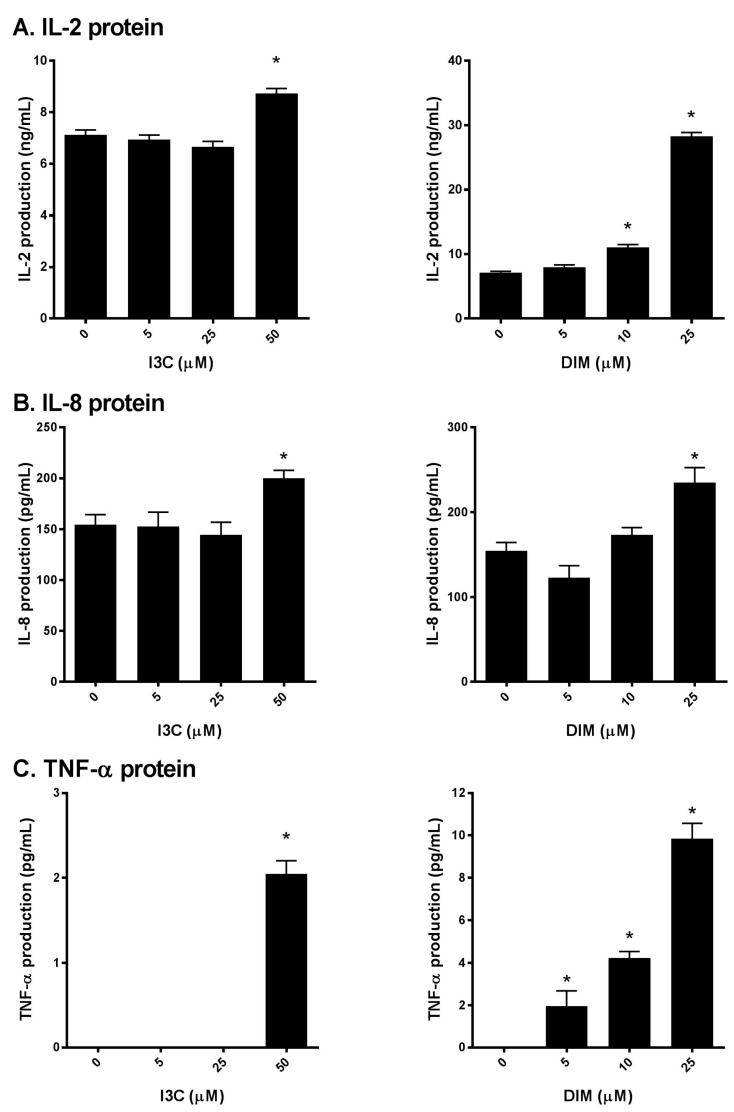
Effects of I3C, DIM on IL-2, IL-8 and TNF-α protein secretion in Jurkat cells. Jurkat cells were treated with 5, 25 or 50 μM of I3C or 5, 10 or 25 μM of DIM for 48 h and then stimulated with PMA + ionomycin for 24 h. Media were harvested and: (**A**) IL-2; (**B**) IL-8; and (**C**) TNF-α protein determined using enzyme linked immunosorbent assay (ELISA). Results expressed as mean ± SD (*n* = 3) from three independent experiments. * indicates significantly different from control at *p* < 0.05 (Two-Way Anova).

Next, to further confirm our initial findings, we examined the production of IL-2, IL-8 and TNF-α secreted into the media following I3C or DIM pretreatment and subsequent stimulation of Jurkat cells with PMA/ionomycin. We found that I3C, at 50 µM, and DIM, mostly at 10 and 25 µM significantly induced IL-2 (Figure 3A), IL-8 and TNF-α (Figure 3B,C) protein levels, suggesting augmentation of the TCR signaling by I3C and DIM leading to pronounced secretion of inflammatory mediators.

### 2.4. I3C and DIM Activate Nuclear Factor κB (NF-κB) Signaling

**Figure 4:**
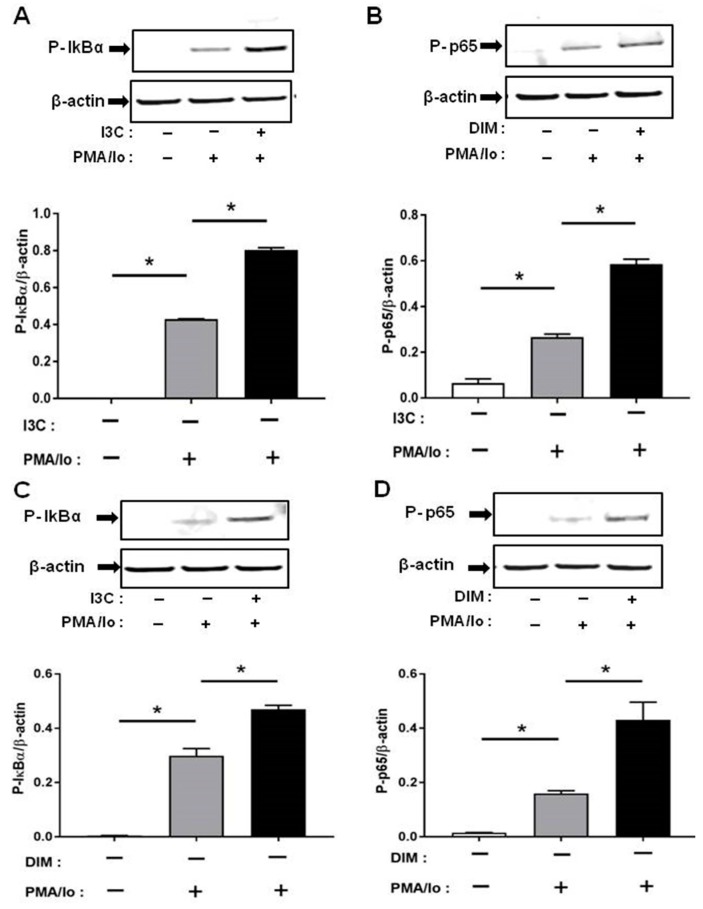
Effects of I3C, DIM on Nuclear Factor κB (NF-κB) activation in Jurkat cells. Jurkat cells were treated with 50 μM of I3C or 10 μM of DIM for 48 h and then stimulated with PMA + ionomycin for 10 or 30 min. Cells were harvested for protein. (**A**,**C**) Phosphorylation levels of Iκ-Bα (ser32); and (**B**,**D**) Phosphorylation levels of Nuclear Factor κB-p65 (NF-κB-p65) (ser536) were determined by western blot analysis. Protein expression was normalized to levels of housekeeping gene (β-actin). The inset shows representative Western blots. * indicates significantly different from control at *p* < 0.05 (One-Way Anova and unpaired *T*-test).

We next investigated the mechanisms underlying the preferential induction of inflammatory cytokines by PMA/ionomycin following I3C/DIM treatment. T cell receptor (TCR) activation is known to result in a rapid release of intracellular Ca^2+^ which in turn stimulates downstream Ca^2+^-sensitive inflammatory signaling pathway [4,5]. Induction of pro-inflammatory cytokines have been suggested to be mediated through activation of NF-κB signaling. We examined if I3C or DIM pretreatment can further enhance PMA/ionomycin-mediated NF-κB signaling. We found that 50 μM of I3C, and 10 μM of DIM induced phosphorylation of IκB-α, and NF-κB-p65 (Figure 4A,B), providing evidence for activation of NF-κB with subsequent pronounced release of pro-inflammatory cytokines.

Phosphorylation was very rapid and was captured at 10 min for p-IκB-α and 30 min for p-NF-κB-p65, following PMA/ionomycin incubation.

### 2.5. I3C and DIM Differentially Induce Nuclear Factor of Activated T-Cells 1 (NFAT1) Dephosphorylation

**Figure 5:**
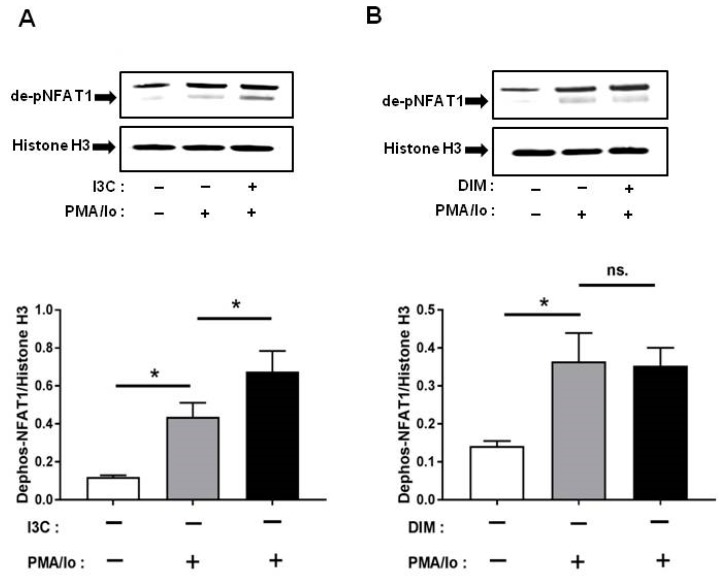
Effects of I3C, DIM on nuclear factor of activated T-cells 1 (NFAT1) dephosphorylation in Jurkat cells. Jurkat cells were treated with 50 μM of I3C or 10 μM of DIM for 48 h and then stimulated with PMA+ionomycin for 10 or 30 min. Nuclear extracts were prepared following cell harvest and dephosphorylation of NFAT1 was analyzed by western blot in: (**A**) I3C-pretreated cells; and (**B**) DIM-pretreated cells. Protein expression was normalized to levels of housekeeping gene (Histone H3). The inset shows representative Western blots. Results expressed as mean ± SD (*n* = 3). * indicate significantly different from control at *p* < 0.05 (One-Way Anova and unpaired *T*-test); and ns., represents not significant.

Other than NF-κB, one of the other major signaling pathways regulated by Ca^2+^ is NFAT [14]. Among the NFAT family, NFAT1 is the most abundant in T lymphocytes [15]; therefore, we examined whether I3C and DIM pretreatment can induce NFAT1 signaling further following TCR activation. We found that pretreatment with I3C at 50 µM can significantly induce NFAT1 dephosphorylation (and activation) within 10 min of stimulation with PMA/Ionomycin. In contrast, DIM pretreatment could not induce NFAT1 activation to the same extent (Figure 5A,B).

## 3. Discussion

I3C and its principal byproduct DIM have been reported to possess numerous health benefits, including anti-inflammatory and anti-carcinogenic properties [30,31,32,33,34,35,36,37,38,39,40,41]. Given that T-cells are intricately involved in the regulation of inflammatory responses, our objective was to evaluate whether I3C and DIM can modulate T-cell mediated immunity. T-cell activation can be induced by a combination of different agents. We chose two widely used combination: PMA/anti-CD3 and PMA/ionomycin to stimulate a human T lymphocyte cell line, Jurkat (Clone E6-1). Although CD3 stimulates T-cells through TCR ligation [42,43], PMA and ionomycin work in a different manner, bypassing the TCR pathway. While PMA, a phorbol ester, directly activates protein kinase C theta (PKCθ) [44], ionomycin can promote T-cell activation through the increase of intracellular Ca^2+^ [45]. We observed that these two combinations induced differential pro-inflammatory responses when pretreated with I3C and DIM. Interestingly, compared to PMA/anti-CD3, PMA/ionomycin combination induced significantly higher levels of inflammatory cytokines IL-2, IL-8 and TNF-α expression and secretion. Moreover, I3C and DIM pretreatment, in a dose-dependent manner, further enhanced the inflammatory cytokine expression. However, at the protein level, I3C and DIM, only at higher concentrations, could significantly induce IL-2, IL-8 and TNF-α. This observation led us to further investigate the underlying mechanisms by which I3C and DIM, at pharmacological doses modulate immune responses in T cells. Since the difference in the two stimulation cocktails was the presence of ionomycin, which acts through elevation of intracellular calcium, we next focused on examining Ca^2+^-sensitive downstream inflammatory signaling proteins involved in these immune responses.

Nuclear factor (NF)-κB plays an important role in T-cells by regulating and initiating an inflammatory response, leading to proliferation, differentiation and survival of T cells [46]. Previous studies have reported that an increase in Ca^2+^ positively regulates NF-κB activation and inhibition of calcium can block NF-κB activity [41,42]. Since we observed a profound increase in pro-inflammatory cytokine levels following TCR-induction of I3C/DIM pre-treated Jurkat cells, this observation prompted us to examine whether I3C and DIM pretreatment enhanced NF-κB signaling. Our results showed that I3C and DIM pretreatment, markedly stimulated phosphorylation of I kappa B-α (IκBα), a protein known to retain the inactive form of NF-κB in the cytosol [12]. Moreover, the treatments induced p65 phosphorylation at ser^536^, which is predominantly cytosolic, suggesting activation of NF-κB signaling pathway. We also examined whether I3C and DIM can similarly induce another downstream pathway involved in the regulation of pro-inflammatory cytokine release—NFAT signaling. However, although I3C pretreatment could significantly induce TCR-mediated NFAT1 dephosphorylation, DIM pretreatment failed to cause similar effect. We speculate that the differential effects observed could be due to the degree of calcium flux triggered by I3C and DIM. Unlike NF-κB, a more sustained calcium influx is needed to induce NFAT1 [44], which produced a differential effect on NFAT1 activation mediated by I3C and DIM pretreatment.

Of note, our results on phosphorylation of IκBα differ from others, such as those reported by Michijima et al. and Takada et al. [47,48]. The discrepancy can be explained based on a number of factors, such as experimental design, doses used, selection of time points and choice of stimulating agents. Michijima et al. in addition to using a higher dose of I3C (100 µM), examined phosphorylation event at a much longer incubation time period [47], which may have resulted in failure to capture the phosphorylation peak. Takada et al. investigated the effect of TNF-α on phosphorylation of IκBα. TNF-α is a pleiotropic cytokine that binds to its receptor to activate diverse downstream pathways leading to inflammation [49], whereas PMA/ionomycin has been widely used for studies related to T-cell activation and dissecting the TCR pathway. Once again we point out that differences in our results compared to those reported earlier [48] are likely due to issues related to differences in experimental design and the signaling pathways studied. Further research is needed to address these issues and decipher the mechanisms of action.

In summary, we found that I3C and DIM pretreatment, at pharmacological concentrations, can induce pro-inflammatory cytokine release through activation of NF-κB and NFAT1. The increased inflammatory response observed may be due to priming of the T-cells by I3C and DIM, rendering the cells more responsive to TCR stimulation. This latter event can trigger activation of downstream NF-κB, and NFAT1 signaling, ultimately leading to a pronounced induction of inflammatory cytokines. Currently, I3C and DIM are being promoted as chemopreventive agents and are commercially available as dietary supplements for consumer use. Several studies, including ours [27,28,29,50], have reported gastrointestinal distress, nausea and vomiting as common side effects following administration of I3C/DIM. Consumers need to be aware that the notion of “more is better” is not applicable in this regard and can sometimes lead to deleterious consequences. Our observation in this study, therefore, serves as a caution for the use of these food bioactives in the treatment of inflammatory diseases, as use of I3C or DIM at pharmacological concentrations may aggravate the underlying inflammation in a vulnerable population and worsen disease outcome.

## 4. Materials and Methods

### 4.1. Reagents and Antibodies

I3C, DIM, phorbol-12-myristate-13-acetate -+(PMA), ionomycin, dimethyl sulfoxide (DMSO) and CCK8 kit were purchased from Sigma-Aldrich (St. Louise, MO, USA). TRIzol, AffinityScript Multiple Temperature cDNA Synthesis kit, and TaqMan real-time PCR primers and probes were obtained from Life Technologies/ThermoFisher Scientific (Waltham, MA, USA). p-IκBα (ser32), p-NF-κB-p65 (ser536), β-actin, histone H3 and NFAT1 antibodies were purchased from Cell Signaling Technology (Denver, MA, USA). NE-PER Nuclear and Cytoplasmic Extraction Kit was purchased from ThermoFisher Scientific (Waltham, MA, USA).

### 4.2. Cell Culture and Treatment

Jurkat (clone E6-1) cells, (American Type Culture Collection (ATCC), Manassas, VA) were cultured in RPMI-1640 Medium (Invitrogen, Carlsbad, CA, USA) supplemented with 10% fetal bovine serum (FBS; Invitrogen) and 100 U/mL penicillin, 100 mg/mL streptomycin (ThermoFisher Scientific, Grand Island, NY, USA). Cells at a density of 0.3 × 10^6^/mL were plated in T-75 flasks overnight. Twenty-four hours later, treatment began. Cells were treated with 10, 25 or 50 μM for I3C and 5, 10 or 25 μM for DIM (DMSO as vehicle) for 48 h and the medium containing test compounds was replaced every 24 h. Forty-eight hours later, cells were stimulated with a mixture of PMA (25 ng/mL) and ionomycin (1 μg/mL) for various time points and harvested for real-time PCR (RT-PCR) and western blot assays. For enzyme linked immunosorbent assay (ELISA), similar experiments were carried out and supernatants were harvested. Secreted cytokine levels in the supernatant were normalized to protein concentrations (BCA assay).

### 4.3. Cell Viability Assay

Jurkat cells (ATCC, Manassas, VA, USA) were plated at a density of 4 × 10^5^ cells/mL in a 96 well plate overnight. Cells were treated with I3C or DIM at different doses for 48 h and cell viability was assessed using CCK8 kit following manufacturer’s instructions.

### 4.4. Real-Time PCR Analysis

Total RNA was isolated using TRIzol reagent (ThermoFisher Scientific, Grand Island, NY, USA) and cDNA was synthesized using AffinityScript Multiple Temperature cDNA Synthesis kit (AgilentTechnologies, Santa Clara, CA, USA). Real-time PCR was carried out using a TaqMan Fast Universal PCR Master Mix on a 7900HT FAST real-time PCR System (Applied Biosystems, Foster City, CA, USA). Relative mRNA fold changes to control were calculated using the comparative *C*T2^−ΔΔ*C*T^ cycle (Δ*C*T) method following manufacturer’s directions. Human TATA–box binding protein (TBP) was used as an endogenous control for all gene expression analysis calculation.

### 4.5. Western Blot Analyses

Total protein was extracted in a lysis buffer (RIPA) containing protease and phosphatase inhibitors for preparation of whole cell lysates by centrifugation at 14,000 rpm at 4 °C for 15 min. Protein concentration was determined using the BCA protein assay (Pierce Biotechnology, Rockford, IL, USA). For western blotting, protein (20 μg) was loaded on 10% Bis-tris Nupage gels, transferred onto nitrocellulose membrane using the iBlot gel transfer apparatus (GE Healthcare, Munich, Germany). The blot was blocked with Licor Blocking buffer for an hour at room temperature and then washed 4 × 10 min with 1× PBS/Tween 20. Then the blot was incubated in Licor secondary antibodies for 1 h, followed by washing 4 × 10 min at room temperature. Proteins were analyzed using an Odyssey Infrared Imaging System (LI-COR Biosciences, Lincoln, NE, USA). For NFAT staining, nuclear extracts were separated from total cell lysates using NE-PER nuclear extraction kit and western blot was carried out as previously described.

### 4.6. Determination of IL-2, IL-8, TNF-α Proteins by Enzyme-Linked Immunosorbent Assays

Jurkat cells were plated in T75 flasks at a cell density of 0.3 × 10 ^6^/mL overnight. The following day, cells were treated with I3C (10, 25, 50 µM) or DIM (5, 10, 25 µM) for 48 h (within the 48 h time frame, cells were replenished with fresh media and I3C/DIM after the first 24 h). Following 48 h incubation, cells were stimulated with PMA (25 ng/mL) and ionomycin (1 µg/mL) for 24 h and the supernatants were harvested to detect protein levels of IL-2, IL-8 and TNF-α using commercially available ELISA kits (Biolegend, San Diego, CA, USA).

### 4.7. Statistics

Graph Pad Prism 6 program (2012, Graph Pad Software, La Jolla, CA, USA) was used for statistical analysis of data. Data were analyzed using one-way ANOVA and unpaired *T*-test. Gene expression results are expressed as mean ± SD relative to vehicle control. Data are representative of 3 independent experiments.
